# Migration context of emergency department patients and patterns of their in- and outpatient healthcare utilization – analysis of three INDEED study centers in Berlin

**DOI:** 10.1186/s12889-025-22023-9

**Published:** 2025-02-28

**Authors:** Freddy Irorutola, Anna Schneider, Katharina Verleger, Dana Abdel Fatah, Samipa Pudasaini, Antje Fischer-Rosinsky, Anna Slagman, Stephanie Roll, Thomas Keil, Martin Möckel, Liane Schenk

**Affiliations:** 1 Charité – Universitätsmedizin Berlin, Emergency and Acute Medicine (CVK, CCM), Berlin, Germany; 2 Charité – Universitätsmedizin Berlin, Institute of Medical Sociology and Rehabilitation Science, Berlin, Germany; 3 Humboldt-Universität zu Berlin, Berlin Institute for Empirical Integration and Migration Research (BIM), Berlin, Germany; 4 Charité – Universitätsmedizin Berlin, Institute of Social Medicine, Epidemiology, and Health Economics, Berlin, Germany; 5 University of Würzburg, Institute of Clinical Epidemiology and Biometry, Würzburg, Germany; 6 Bavarian Health and Food Safety Authority, State Institute of Health I, Erlangen, Germany

## Abstract

**Background:**

Increasing sociocultural diversity has implications for emergency department (ED) care. Individuals with a migration context face challenges that can manifest as barriers in healthcare access and use. Therefore, our aim was to examine differences in healthcare delivery for and utilization of ED patients in Germany regarding their migration context.

**Methods:**

We utilized routine healthcare data from the INDEED project. Patient records from three EDs in Berlin, Germany, from 2016 were linked with associated outpatient treatment data spanning 2014 to 2017. Using an onomastic approach, patients were assigned to one of nine regions of origin (refers to “migration context”) based on their names in addition to citizenship. Demographic and clinical data were compared between patients with and without a presumed migration context. Regression analyses were conducted to determine the association of migration context on triage category, hospital admission, frequent ED use (more than two visits within one year), and the number of outpatient presentations, adjusting for sex, age in years and multimorbidity (more than three different diagnoses over three calendar quarters within four consecutive calendar quarters before the first ED visit).

**Results:**

In total, 123 572 (49 003 (40.2%), 74 569 (59.8%) without presumed migration context) cases were examined. ED patients with a presumed migration context were younger and more often male than those without. Adjusted regression analyses demonstrated that the overall migration context was associated with a slightly higher likelihood of more urgent triage categorization (adjusted odds ratio: 1.03; 95%-confidence interval: 1.01–1.04), while it was also associated with fewer hospital admissions after the ED stay (adjusted odds ratio 0.92; 95%-confidence interval 0.90–0.96), being a frequent ED user (1.22; 1.15–1.30), and fewer contacts with outpatient healthcare services (exponentiated estimates 0.86; 0.85–0.86).

**Conclusion:**

Our routine data analysis highlighted differences in healthcare pathways between ED patients with and without a presumed migration context in Germany. The likely complex and multifactorial reasons behind these disparities warrant further investigation, preferably by prospective studies. Understanding these factors can enhance the promotion of healthcare provision that is more sensitive to a diverse society.

## Introduction

### Background

Dealing with sociocultural diversity presents a challenge in the German healthcare system, leading to differences in care for patients with a migration context [1–5]. We conceptualize “migration context” as a space of experience of individuals that is constituted by life-historical imprints of the context of origin, a cross-border relocation of the center of life - the migration event -, the specific conditions and experiences in the immigration context and transnational experiences [6].

Several studies have shown that migrant patients are frequently confronted with unequal access to healthcare services, longer waiting times, fewer diagnostic procedures, shorter consultation durations, and generally lower satisfaction with the treatment received [2, 7–9].

To date, research in EDs on healthcare provision for patients with a migration context has largely focused on their usage behavior, often revealing that patients with a migration context are more often categorized as “frequent users” [4]. Additionally, there is evidence in Germany suggesting that a migration context is associated with a lower utilization of outpatient healthcare services including visits to specialists, rehabilitation services, and preventive measures such as cancer screening programs [9]. These disparities are mirrored in international findings, where being part of a minority ethnic group has been shown to affect numerous aspects of emergency care: less urgent triage categorization, reduced administration of pain relief medication, fewer treatment interventions, less diagnostic testing, an increased likelihood of leaving the ED without receiving care, and a lower satisfaction with the overall patient experience in the ED [3, 10].

Given their critical role in identifying and treating serious illnesses, EDs shoulder a substantial responsibility to ensure equal, unbiased access and treatment. However, they often grapple with pressures such as crowding and time constraints, which can amplify biases in patient treatment based on factors such as migration context, especially during times of resource scarcity [11, 12]. Given the available evidence, the urgent need for diversity-sensitive care in ED settings has become clear. To actively reduce biases and foster equitable care, a comprehensive understanding of the cross-sectoral care patterns underlying these disparities is important. In Germany, there is currently a lack of studies on cross-sectoral care patterns in the context of ED treatment, particularly for individuals with a migration context.

### Objectives

Utilizing an onomastics-based methodology alongside routine dataset analysis, our research aims to analyze potential disparities in emergency care between patients with and without a migration context in terms of triage categorization, inpatient admission and the number of frequent ED users. We further aim to conduct a comparative examination of outpatient visits prior to the index ED visit.

## Methods

### Study design and data management

This investigation is a secondary data analysis and patient cohort study utilizing routine data derived from the INDEED project [13].

The INDEED project, funded by the German Innovation Fund (grant number: 01VSF16044), aimed to analyze the “utilization and trans-sectoral care patterns of patients in emergency care structures in Germany”. This goal was achieved through a pseudonymous, individual-level linkage of routine data from 16 EDs in Germany from 2016 with outpatient care data from 2014 to 2017. All ED patients who were of legal age (≥ 18 years) in 2014 and who were covered by statutory health insurance were included.

A detailed description of data processing within the INDEED project was published previously [13].

Our investigation utilized an onomastics-based approach to identify ED patients with and without a migration context. The onomastic method implemented by Humpert and Schneiderheinze infers specific regions of origin from linguistic insights derived from name research [14]. The onomastic method has been applied in Germany in various contexts, including by the Expert Council of German Foundations for Migration and Integration (SVR) and in a survey conducted by the Federal Institute for Population Research (BiB) [15–17]. Based on the patients’ names, categorization was performed using program-driven coding, matching with known codes, verification and manual allocation through visual inspection in nine regions of origin. The first and last names of patients were only available for three out of the 16 study centers of the INDEED project. Therefore, only these three EDs were considered in this analysis. One person proficient in the onomastics procedure allocated one of the nine predefined region codes (see description in Sect. 2.6 Variables) to each patient in the dataset. To relink region codes and further variables per patient, first and last names were deleted from the previous dataset, and information on the region codes was added to the original dataset via the patient pseudonym.

### Variables

Migration context: Nine regions of origin were preselected by researchers with regard to the most common regions of origin for migrants registered in Berlin in 2016 [18]. A region of origin as defined in our study could encompass multiple countries to take into account historical shifts in national borders, migration flows and associated linguistic similarities within specific regions comprising multiple countries.

The assignment and categorization of countries of origin followed two guiding principles. First, the representation logic for origin groups aimed to reconstruct the most frequently represented countries of origin in the Berlin study region in 2016—namely Turkey, Poland, Russia, Italy, Syria, Lebanon, Bulgaria, and the USA. This approach was based on the assumption that these countries would also be reflected in the population of the emergency department and that the case numbers would be sufficient to allow for origin-specific analysis. Second, the onomastic logic addressed the challenge of clearly assigning certain names to specific countries of origin. For instance, within the onomastics procedure, individuals with Arabic names—excluding those from the Maghreb states (Algeria, Morocco, and Tunisia)—could only rarely be linked to specific nationalities with adequate accuracy. This limitation arises from the shared language, naming conventions, and the historically and politically influenced national borders of the region, which often do not correspond to dialects or name distributions.

Due to the small number of cases available for isolated analyses and financial constraints, all remaining countries of origin were grouped into a single category.

The regions used in our analysis included Germany, Bulgaria, Italy, Poland, former Soviet Union (Belarus, Ukraine, Russia), Turkey, Vietnam, Anglo-Saxon states (Australia, Canada, Ireland, New Zealand, United Kingdom, United States of America, South Africa), Arabic states (Egypt, United Arab Emirates, Iraq, Jordan, Kuwait, Lebanon, Libya, Oman, Palestine, Saudi Arabia, Syria, Yemen) and other or undefined regions. Information on the region of origin from the onomastics procedure was additionally verified by information from the documented citizenship in routine data, which was collected during the administrative registration in the ED. All patients who did not have a migration context according to the onomastics method but had non-German citizenship recorded in routine data were categorized into the respective region of origin corresponding to their citizenship.

We decided against relying solely on nationality for categorization for several reasons. Nationality does not adequately capture all segments of the migrant population, such as naturalized migrants who acquire German citizenship after naturalization or (late) resettlers from the former Soviet Union, Poland, and Romania, who are granted German citizenship upon immigration. Additionally, nationality is often recorded inaccurately in emergency departments, as it is irrelevant for billing purposes and the default entry is typically set to “German.” For these reasons, the exclusive use of nationality is insufficient for reliably identifying migration contexts.

There were 35 missing values for which categorization could not be made (e.g., due to missing names or the lack of plausible categorization).

Outpatient presentations: To estimate the number of outpatient visits, services recorded during the study period from 2014 to 2017 were included based on the billing code of the *Einheitlicher Bewertungsmaßstab* (Uniform Value Scale for Physicians). A selection of services for the one-year period before the index ED presentation in 2016 was conducted. All services per day, specialty and patient were counted as one visit. Outpatient services not directly related to a visit, such as flat-rate charges for letters and postage, basic charges for specialist physicians, and laboratory testing, were excluded. Furthermore, all services that were provided as outpatient services in the participating EDs were excluded.

If no health insurance data were available over the entire four-year period, a missing value was assigned for the respective patients. In these cases, it was assumed that the reason for the missing values was the inability to link the ED data with the health insurance data. If health insurance data were available but there was no outpatient visit in the year before the ED visit according to the above criteria, it was assumed that the patients had not made any outpatient visits.

With these criteria, there were 16 593 patients with no available health insurance data (16.8% of all patients; 12.5% of patients without a migration context, 23.4% of patients with a migration context). Poisson imputation, was performed to address these missing data [19]. A total of five imputed datasets were generated using the mice package in R [20]. The algorithm was allowed a maximum of 20 iterations for the imputation process. To ensure the reproducibility of our results, a random seed of 123 was set prior to imputation.

Frequent users: The categorization into frequent users was based on the number of visits to the three examined EDs in 2016. Three categories were defined: occasional users (1–2 visits), frequent users (3–9 visits), and very frequent users (10 or more visits) [21]. For regression analyses, two groups were defined (occasional users with 1–2 visits and frequent users with more than 2 visits). There were no missing values for the variable frequent user.

Hospital admission: The variable hospital admission categorized patients into groups that had either been admitted to the hospital or discharged after the respective ED stay in 2016. This variable had no missing values.

Triage category: The urgency level in the ED was assessed using the Manchester Triage System (MTS). It is divided into five categories, with “1” representing the most urgent category [22]. For regression analyses, patients were grouped into urgent patients (categories 1–3) and less urgent patients (categories 4 or 5).

There were 6 342 missing values (5.1% of the cases). Regression analyses were conducted based on complete cases.

Multimorbidity: Multimorbidity was defined as having at least three different diagnoses over three calendar quarters within four consecutive calendar quarters before the quarter of the patient’s first ED visit. Three categories were established (yes: multimorbidity conditions met, no: multimorbidity conditions not met and at least one diagnosis assigned; unspecified: all diagnoses missing) [23].

### Ethics

Ethical approval for the INDEED project was obtained from the Ethics Committee of Charité – Universitätsmedizin Berlin, including additional approval for the implementation of the onomastics procedure for data from the three study Emergency Departments (EDs) in Berlin (EA4/086/17). The study is registered in the German Clinical Trials Register (DRKS00022969, Date of registration: October 22, 2020) and was conducted in accordance with the Declaration of Helsinki.

### Statistical methods

Statistical analyses were conducted using the R Project for Statistical Computing version 4.3. Differences in demographic and clinical data between patients with and without migration context were evaluated using the chi-square test for categorical variables and t-tests for continuous variables.

In INDEED, we followed an explorative analysis approach to examine various research questions rather than conducting confirmatory analyses with confirmatory hypotheses. Thus, we did not adjust for multiple testing and all results are interpreted exploratively.

Our analyses were performed both at the case level (cases defined as *visits* to the ED, some patients visited the ED more than once in 2016) and at the patient level. The latter was conducted to examine the utilization of outpatient health services and for frequent users.

To identify factors that are potentially associated with triage category, inpatient admission and classification as frequent user Generalized Linear Mixed Models (GLMMs) were performed, incorporating the center as a random effect to account for clustering. In multiple regression analyses migration context was treated as the independent variable with adjustments made for potential confounders sex, age in years and multimorbidity (i.e. at least three coexisting conditions). To assess factors that are potentially associated with the number of outpatient visits, a Poisson regression was performed.

In all regression models, analyses with specific regions of origin were conducted in addition to the overall analyses of patients with and without a migration context.

Marginal R-square and pooled McFadden’s R-square statistics were calculated for each model to assess goodness of fit in logistic and linear regression analyses, respectively. For the GLMMs, the Intraclass Correlation Coefficient (ICC) was additionally determined to assess the proportion of variance explained by the random effect (center). From these models odds ratios and 95% confidence intervals are presented.

## Results

### Selection of the study population

**Fig. 1:**
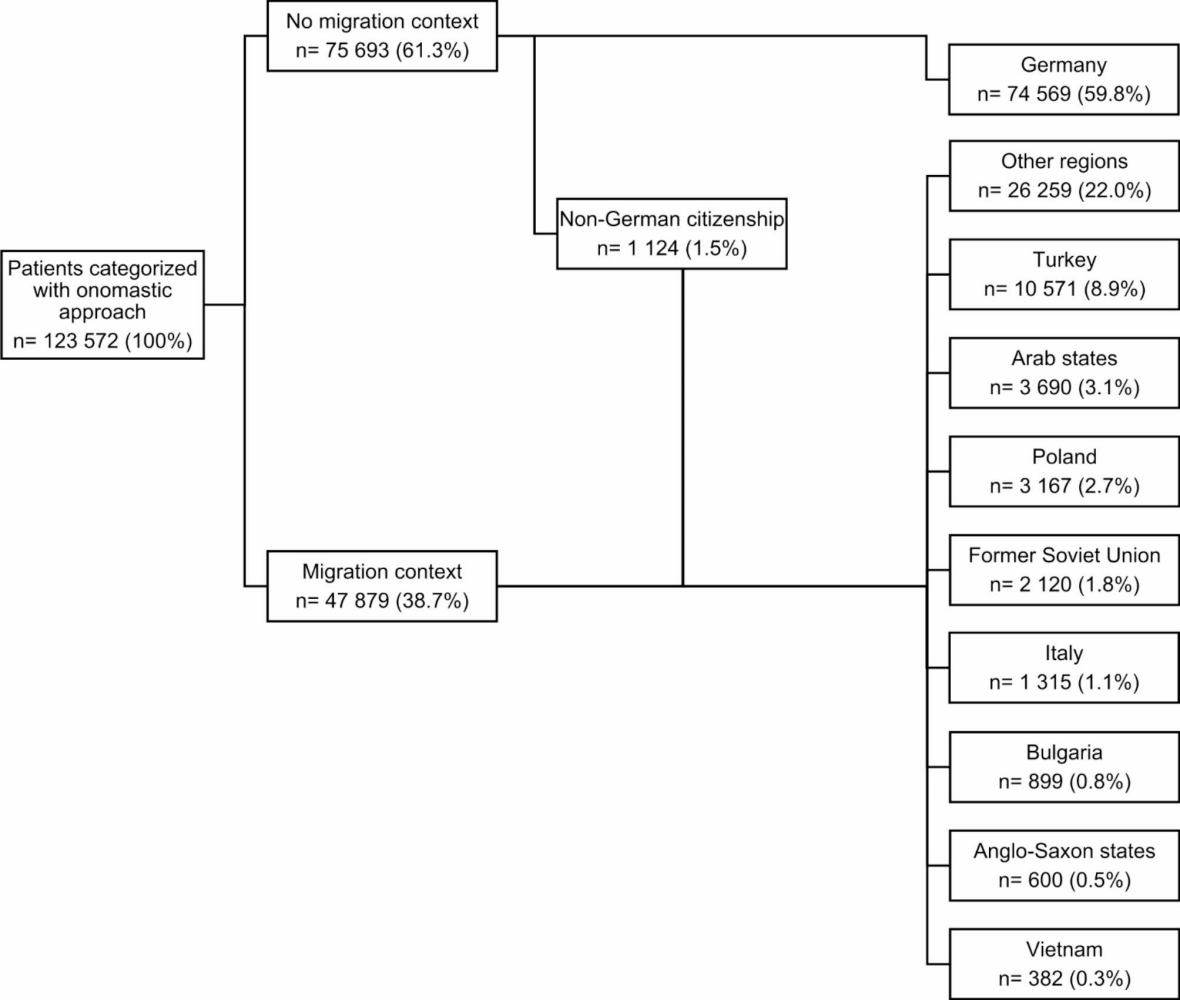
Distribution of the cases based on patients’ assumed origin (based on onomastic procedure and citizenship)

In total, 123 572 cases were examined (Fig. 1). Of these, 47 879 (38.7%) were classified as having a migration context based on their names. In 1 124 (1.5%) cases, the citizenship was not German, thus an assignment to the respective region corresponding to that citizenship was made. This resulted in 49 003 (40.2%) cases with a migration context. In 22.0% (26 259), no specific region of origin was assigned because they did not belong to the 9 most common regions (“other regions”). The second most frequent assignment among cases with an assumed migration context was Turkey, accounting for 8.9% (10 571).

### Demographics, clinical variables and healthcare utilization

#### Description of demographic, clinical variables and healthcare utilization

Patients with a migration context were younger and more often male than patients without a migration context (Table 1).

**Table 1:** Description of demographic and clinical characteristics of ED patients with and without a migration context

	**ED patients** **with** **migration context** *N* = 49 003	**ED patients** **without** **migration context** *N* = 74 569	*p*-Value
**Age in years** (mean ± SD) NA = 10 (< 0.01%)	40.8 ± 16.0	54.8 ± 20.4	< 0.001
**Sex [n (%)]**			< 0.001
Male	24 668 (50.3%)	36 077 (48.4%)	
Female	24 335 (49.7%)	38 492 (51.6%)	
**Manchester Triage Category [n (%)]** NA = 6 342			< 0.001
1 (immediate)	325 (0.7%)	827 (1.2%)	
2 (very urgent)	5 108 (10.9%)	10 778 (15.3%)	
3 (urgent)	21 187 (45.1%)	31 883 (45.3%)	
4 (standard)	18 817 (40.1%)	25 303 (36.0%)	
5 (nonurgent)	1 471 (3.1%)	1 535 (2.2%)	
**Multimorbidity [n (%)]**			< 0.001
Yes	25 404 (51.8%)	52 018 (69.7%)	
No	9 641 (19.7%)	12 149 (16.3%)	
Not specified	13 958 (28.5%)	10 402 (13.9%)	
**Frequent ED users [n (%)]**			0.400
1–2 visits	41 603 (84.9%)	63 512 (85.1%)	
3–9 visits	6 916 (14.1%)	10 337 (13.9%)	
≥ 10 visits	484 (1.0%)	720 (1.0%)	
**Inpatient admission [n (%)]**			< 0.001
Yes	9 642 (19.7%)	23 407 (31.3%)	
No	39 361 (80.3%)	51 162 (68.7%)	
**Number of outpatient service contacts one year before ED visit** (mean ± SD) NA = 16 593 patients	5.1 ± 10.6	7.8 ± 13.4	< 0.001

Abbreviations: ED = emergency department, SD = standard deviation, NA = Not available

Patients with a migration context received less urgent triage categories, were admitted less frequently as inpatients, and had fewer contacts with outpatient healthcare services in the year preceding the ED visit. No relevant differences were observed regarding the distribution of frequent users between patients with and without a migration context (Table 1).

#### Triage

Overall, the migration context was associated with a small increase in the likelihood of more urgent triage categorization in the multivariable regression analysis (Table 2). However, analyses of specific regions of origin revealed variations within the different regions: the probability of an urgent triage was more often given to patients with origin from Poland and Arabic states, whereas no differences where observed for patients with origins from other regions. Increasing age and the presence of multimorbidity also led to an urgent triage level. Sex was not associated with triage category.

**Table 2:** Associations of the outcome urgent (versus less urgent) triage categorization with sociodemographic factors and Multimorbidity among 123 569 ED patients (Generalized linear mixed Models)

Characteristics	Odds Ratio	95% confidence interval	*p*-value
Migration context (vs. none)	1.03	1.01, 1.04	0.009
Bulgaria	1.10	0.96, 1.25	0.166
Italy	0.95	0.85, 1.06	0.398
Poland	1.11	1.03, 1.20	0.003
Former Soviet Union	1.00	0.95, 1.13	0.426
Turkey	1.03	0.95, 1.04	0.886
Vietnam	0.93	0.75, 1.13	0.458
Arabic states	1.17	1.10, 1.26	< 0.001
Anglo-Saxon countries	1.10	0.94, 1.30	0.244
Other migration context	1.03	1.00, 1.06	0.091
Age in years	1.01	1.01, 1.01	< 0.001
Female sex (vs. male/diverse/not specified)	1.00	0.98, 1.02	0.871
Multimorbidity (versus none)	1.14	1.10, 1.17	< 0.001
Unspecified multimorbidity ( versus none)	0.92	0.89, 0.96	< 0.001

Marginal R-square = 0.01, ICC = 0.01, *n* = 123 549. Adjusted for all presented variables with center as a random effect. Unadjusted odds ratio for migration context vs. none: 0.85 (0.83–0.87), *p* < 0.001

#### Inpatient admission after an ED visit

A migration context was associated with a lower likelihood of inpatient admission. However, marked differences between specific regions of origin were observed in multivariable analyses. Vietnam, Arabic states and Anglo-Saxon countries as regions of origin had no relevant influence on the likelihood of inpatient admission after the ED stay, while Poland and Bulgaria where the only regions in which inpatient admission was more probable (Table 3). Sex also had a significant influence: women were less likely to be admitted to the hospital than men. Other factors that increased the likelihood of inpatient admission included increasing age, the presence of multimorbidity, and more urgent triage categorization (Table 3).

**Table 3:** Associations of inpatient admission after the ED stay (as outcome, vs. no admission) with sociodemographic factors and Multimorbidity (Generalized linear mixed Models)

Characteristics	Odds Ratio	95% confidence interval	*p*-value
Migration context (vs. none)	0.92	0.90, 0.96	< 0.001
Bulgaria	1.20	1.01, 1.42	0.040
Italy	0.80	0.70, 0.93	0.003
Poland	1.40	1.30, 1.53	< 0.001
Former Soviet Union	0.87	0.78, 0.98	0.016
Turkey	0.80	0.76, 0.85	< 0.001
Vietnam	1.09	0.85, 1.40	0.488
Arabic states	0.92	0.84, 1.00	0.062
Anglo-Saxon states	1.04	0.86, 1.27	0.674
Other migration context	0.93	0.90, 0.97	< 0.001
Age in years	1.03	1.03, 1.03	< 0.001
Female (vs. male/diverse/not specified)	0.75	0.74, 0.78	< 0.001
Multimorbidity (versus none)	1.23	1.18, 1.28	< 0.001
Unspecified multimorbidity (versus none)	0.64	0.61, 0.68	< 0.001

Marginal R-square = 0.14, ICC = 0.01, *n* = 123 548. Adjusted for all presented variables with center as a random effect. Unadjusted odds ratio for migration context vs. none: 0.54 (0.52–0.55), *p* < 0.001

#### Frequent ED users

Having a migration context was overall associated with frequent ED use. Differentiating into regions of origin, Italy, the former Soviet Union, Anglo-Saxon states and Vietnam were, however, not associated with frequent ED use. Furthermore, classification into the frequent user group was more likely with increasing age, the presence of multimorbidity and male sex (Table 4).

**Table 4:** Associations of the outcome frequent ED use (vs. not) with sociodemographic factors und Multimorbidity status (Generalized linear mixed Models)

Characteristic	Odds Ratio	95% CI	*p*-value
Migration context	1.22	1.15, 1.30	< 0.001
Bulgaria	1.49	1.05, 2.11	< 0.004
Italy	0.99	0.71, 1.38	0.878
Poland	0.98	0.79, 1.22	< 0.001
Former Soviet Union	1.05	0.83, 1.33	0.904
Turkey	1.18	1.06, 1.31	< 0.001
Vietnam	0.50	0.22, 1.11	0.055
Arabic states	1.61	1.35, 1.91	< 0.001
Anglo-Saxon states	1.12	1.06, 2.48	0.116
Other migration context	1.01	1.03, 1.22	< 0.001
Age in years	1.01	1.01, 1.01	< 0.001
Female (vs. male/diverse/not specified)	0.79	0.79, 0.89	< 0.001
Multimorbidity (versus none)	1.70	1.55, 1.87	< 0.001
Unspecified multimorbidity (versus none)	0.81	0.72, 0.91	< 0.001

Marginal R-square = 0.03, ICC = 0.01, *n* = 98 867. Adjusted for all presented variables with center as a random effect. Unadjusted odds ratio for migration context vs. none: 1.00 (0.94–1.06), *p* > 0.9

#### Outpatient presentations

Having a migration context resulted in fewer contacts with outpatient services within one year before the index ED visit in the adjusted model, holding all other variables constant. This association was even stronger for several specific regions of origin including Bulgaria and Arabic states. Furthermore, older age, multimorbidity and female sex also resulted in more contacts with outpatient services (Table 5).

**Table 5:** Associations between the number of contacts with outpatient healthcare services and sociodemographic factors as well as Multimorbidity (adjusted Poisson regression analysis)

Characteristic	Exponentiated Estimate	95% CI	*p*-value
Migration context	0.86	0.85, 0.86	< 0.001
Bulgaria	0.65	0.61, 0.69	< 0.001
Italy	0.84	0.82, 0.87	< 0.001
Poland	0.83	0.82, 0.85	< 0.001
Former Soviet Union	0.90	0.88, 0.92	< 0.001
Turkey	0.85	0.84, 0.86	< 0.001
Vietnam	0.84	0.80, 0.89	< 0.001
Arabic states	0.77	0.75, 0.78	< 0.001
Anglo-Saxon countries	0.96	0.91, 1.00	< 0.001
Other migration context	0.87	0.86, 0.88	< 0.001
Age in years	1.01	1.01, 1.01	< 0.001
Female (vs. male/diverse/not specified)	1.06	1.05, 1.07	< 0.001
Multimorbidity (versus none)	3.01	2.98, 3.04	< 0.001
Unspecified multimorbidity	2.12	2.09, 2.15	< 0.001

Pooled McFadden’s R-square = 0.19, *n* = 98 881. Adjusted for all presented variables, Unadjusted exponentiated estimate migration context vs. none: 0.72 (0.72- -0.72), *p* < 0.001

## Discussion

### Main findings

Our analysis of secondary data from three large EDs in Berlin, Germany, provided evidence of differences in emergency care depending on the migration context of the patients. Patients with a migration context showed a small increase in the likelihood of being assigned a more urgent triage category. They were also less frequently admitted for inpatient treatment after their ED stay and were more often frequent users, even when relevant sociodemographic and health-related characteristics were controlled for. In the year before their initial ED visit, patients with a migration context made less use of outpatient care services. However, there were marked differences in the examined aspects of care within the migrant population with respect to specific regions of origin.

#### Frequency of ED and outpatient services use

While no group differences of ED use between patients with and without a migration context were observed, our results showed that a migration context increased the likelihood of being a frequent user when adjusting for age, sex, and multimorbidity. Previous studies on similar topics have reported inconsistent results. In Germany, one primary study conducted in three hospitals in Berlin, published more than 20 years ago, suggested that patients with a migration context made use of emergency services more frequently than those without [24]. In contrast, another primary 2012 study from Berlin, which included the collection of sociodemographic information, revealed no difference in the utilization of EDs between people with a Turkish or other migration context and those without [25]. More recent investigations from Berlin have been lacking.

Similarly diverging results are noticeable in the European context. For instance, studies from Spain, England, Switzerland, Denmark, Italy, and Norway also indicated more frequent usage or a greater proportion of frequent users in populations with a migration context [26–39]. Sandvik et al. from Norway and Buron et al. from Spain, however, indicated that people with a migration context had fewer contacts with EDs than those without a migration context [40, 41].

Additionally, we identified a lower number of outpatient presentations in our patient group with a migration context. These findings align with previous studies, which have reported lower outpatient service utilization among patients with a migration context [9, 42].

These differences in utilization patterns are likely complex and multifactorial. Possible reasons for higher ED use and fewer outpatient presentations could range from unequal access opportunities to healthcare services and experiences of discrimination to information deficits and varying healthcare needs [2].

Unequal access opportunities to the healthcare system can result from unequal “system knowledge” [43]. “System knowledge” includes, among other things, knowledge of accepted paths, codes of conduct and invisible “rules of the game” in healthcare. This knowledge, which supports successful navigation through the healthcare system, is conveyed through experience, i.e. depending on the socialization context.

Research has explored how discriminatory behavior by healthcare providers can manifest in their interactions with patients, focusing on the dynamics between healthcare providers and patients. Gerlach et al. found evidence of generalizations, discriminatory actions, and exclusionary behavior in focus group discussions with general practitioners in dealing with patients with a migration context in Germany [44]. Furthermore, focus group discussions with Turkish and Congolese migrants indicate that patients face a lack of intercultural competence and experience misunderstandings, prejudices and discrimination in their utilization of healthcare services [5, 45]. The Afro-Census surveyed racism and discrimination experiences in healthcare in Germany. More than six out of ten respondents experienced discrimination, mainly because of their skin color and other racist reasons [46]. The 2023 report from the National Discrimination and Racism Monitor (*Nationaler Diskriminierungs- und Rassismusmonitor*; NaDiRa) in Germany revealed that racially marked patients change their doctors more often because they felt that their health complaints are not taken seriously [1]. It also showed that patients tend to avoid medical consultations when they anticipate discrimination. Additionally, the report demonstrated that patient names influence appointment scheduling, with patients with Nigerian and Turkish names receiving appointments less frequently.

Regarding healthcare access, possible barriers are diverse. Language barriers, in particular, present a significant barrier to access and to the quality of healthcare once accessed [47]. Barrier-free communication is essential for the proper assessment of healthcare needs, the provision of information, and the development of patient’s health literacy [48]. Experts therefore designate a broad range of language mediation methods and the simplification of provision as central means to break down barriers for patients with language restrictions [49].

Furthermore, residency status creates legal access restrictions for comprehensive healthcare services for Asylum seekers in Germany [50]. Germany’s statutory health insurance (SHI) system, supplemented by private insurance for certain groups, mandates health coverage for all residents. Asylum seekers typically lack SHI coverage for their first 36 months, receiving healthcare under the Asylum Seekers’ Benefits Act (AsylbLG), which covers acute illnesses, maternity care, vaccinations, and preventive check-ups. After 36 months, they gain full SHI benefits, although some federal states, like Berlin, provide near-full SHI access earlier, issuing health insurance cards since January 1, 2016. Decisions on necessary treatments in these cases rest with physicians. Undocumented individuals cannot enroll in SHI but can access emergency care without physicians reporting their status to immigration authorities. Our dataset includes all ED patients in 2016, regardless of insurance or residency status, ensuring comprehensive data on ED visits.

To encapsulate these findings, a potential explanatory approach suggests that individuals with a migration context more frequently face access barriers and experiences of discrimination in the outpatient healthcare sector. These circumstances lead to a reduced utilization of these services, and consequently, patients with a migration context tend to more frequently resort to EDs as their primary source of care [51]. Additionally, inadequate treatment in EDs, such as inappropriate denials of inpatient admission, may also contribute to frequent return visits to the ED. Sr-on et al. demonstrated that misdiagnosis and suboptimal management contribute to an increased frequency of repeat visits among ED patients [52].

#### Inpatient admission and triage categorization

We found that patients with a migration context are admitted less often for inpatient care than patients without a migration context. Our model adjusted for age, sex and multimorbidity showed that having a migration context was associated with a lower likelihood of inpatient admission. Our findings align with those by Borde et al. and Haji Loueian et al. [25, 48]. It is possible that different health needs in both groups influenced the results. Nevertheless, considering the aforementioned findings, it is also plausible that potential differences in inpatient admission are additionally mediated by access barriers and discrimination [1, 5, 44, 45, 47–50].

We found that patients with a migration context were more likely to be categorized as requiring more urgent care in triage in a multivariable model adjusted for age, sex, and multimorbidity. A scoping review on ethnic minorities in the USA, Canada, and Australia indicated that these groups were often triaged as less urgent in EDs. Additionally, a systematic review focusing on studies from Europe revealed that in most included studies migrants tended to visit EDs more often for less urgent reasons [3, 4]. Our finding may suggest variations in how urgency is perceived or assessed for patients with a migration context within the specific healthcare setting studied. It might also point to real differences in health status or severity of conditions presented at EDs, potentially influenced by barriers to accessing preventive or primary care services among migrants. Additionally, the diversity of patients with different migration contexts might mask any potential subgroup differences. The fact that a significant association was observed in the subgroup from Vietnam, indicating lower triage categorization than patients without a migration context, while another significant association pointed to more urgent triage categorization in patients from Poland, underscores this assumption.

### Strengths and limitations

The strengths of our study encompass the inclusion of three large EDs in an urban setting in Germany, the comprehensive coverage of healthcare services use of included adult patients 12 months before the index ED visit in 2016, and the large number of all ED patients who were treated over the course of one year. Since we used routine data, no drop-outs due to non-response distorted results. This is especially relevant since patients with a migration context are considered a difficult to reach population for surveys [53–56].

Our study also has some limitations. First, apart from citizenship, further characteristics that are associated with a migration context and possibly the utilization of EDs were not recorded in the available ED routine data, e.g. residency status, length of stay in receiving country, reasons for migration, migration generation, German language skills and socioeconomic factors, and thus, potential confounding effects of these variables could not be controlled for in our analysis.

Second, citizenship was documented in only two of the three EDs in the present analysis, the default setting for the citizenship variable in the EDs’ documentation system is ‘German’, leading to the assumption that due to documentation bias, frequency of non-German citizenship would be underestimated by using only this information. In addition, we applied an onomastic method to our dataset, which assigned patients to a regional origin group based on their first and last names. Name-based selection processes require reliable information and respective algorithms to unequivocally assign names to countries or regions of origin. However, there are incorrect assignments of regions of origin. In a study, for persons with a German region of origin, 2% of the assignments from the onomastics procedure were incorrect. In particular, for patients with an assumed migration context incorrect assignments were observed in up to one out of four patients [57]. Some names might be present in different regions and cultures and could therefore be incorrectly assigned to one specific region. In his review, Mateos (2007) argued that temporal and regional variations in name distributions, influenced by migration waves, geographic shifts, and cultural naming practices, can render reference lists outdated and lead to misclassification [58]. Names common in one region may not reflect others accurately, and patrilineal naming systems often obscure mixed ethnicities and women’s identities in mixed marriages [58]. Additionally, challenges like transliterations, misspellings, and name changes due to marriage introduce inconsistencies, while variations in naming conventions, such as retaining maiden names or using multiple surnames, further complicate accurate classification [58]. The onomastic method also does not provide insights into the timing of migration or the generation in which migration occurred. As with the variable citizenship, its purpose does not include the description of further relevant differences within migrant groups, such as socioeconomic status, length of stay in the host country, residence status, language skills, accent, skin color, or religious and cultural orientation. Accordingly, for example, the risks of discrimination between and within groups can vary significantly.

Third, we identified a relatively high number of missing values for the variable related to outpatient visits (*n* = 16 593, 16.8% of the patients). However, in our regression analysis, where we investigated outpatient visits as the dependent variable, we addressed missing values through multiple imputation. Fourth, the regression analyses conducted for both the triage and frequent user models yielded low Marginal R-square values (0.01 and 0.03), suggesting a low ability of the included variables to account for the variability observed in the data. Fifth, our analysis does not provide causal explanations for the observed differences between patients with and without a migration context. Sixth, the complex cross-sectoral dataset required extensive preparation and was only fully available late in the project, leading to delays in analysis despite the dedicated efforts of the research team. As a result, the data used in the analyses reflect an earlier time period, which may limit the current relevance of the findings. Seventh, variations in healthcare access for asylum seekers and undocumented individuals, particularly differences in statutory health insurance coverage, may influence both emergency department utilization patterns and the use of outpatient care services, which are not accounted for in our analysis.

Overall, these limitations indicate that precise and multifaceted assessment strategies to correctly capture the population of patients with a migration context are scarce, which in turn complicates adequate basic research and future improvement of healthcare services for them.

## Conclusions

In our study of 123,572 patients in three large EDs in Berlin, Germany, we found no relevant difference in urgency classification during triage between patients with and without a migration context in the overall cohort. However, we identified differences in the use of care structures among patients with compared to those without a migration context. A migration context was associated with frequent ED use, with fewer inpatient admissions after the ED stay and fewer contacts with outpatient healthcare services.

EDs serve as primary contact points for critically ill patients. These departments often face challenges such as crowding and limited treatment time which raises the potential for implicit or explicit biases in decision-making [12]. It is important to uncover such biases and counteract them, given that EDs often represent the final refuge for people in distress.

An important insight highlighted by this study is that patients with a migration context are very diverse. Examining this group could mask potential care disparities within the group.

Further research should include primary prospective studies to delve deeper into the causes of possible differences and consider the diversity of patients with a migration context. To date, no studies in German EDs have examined the connection between experiences of discrimination and disparities in the German healthcare system. A mixed-methods approach would be worthwhile by triangulating reported experiences of discrimination prior and during ED visits. Moreover, it is vital to reflect on and address potential internalized biases to foster diversity-sensitive and discrimination-free emergency care.

## Data Availability

The dataset analyzed during the current study is not publicly available due to the high sensitivity of the clinical data of the patients in the emergency department. However, they are available from the corresponding author upon reasonable request, potentially in aggregated form to ensure that no identification is possible.
